# Evaluating the predictive value of diabetes mellitus diagnosed according to the Chinese guidelines (2020 edition) for cardiovascular events

**DOI:** 10.1186/s13098-022-00906-w

**Published:** 2022-09-26

**Authors:** Zixiang Ye, Yanxiang Gao, Enmin Xie, Yike Li, Ziyu Guo, Peizhao Li, Jingyi Ren, Jingang Zheng

**Affiliations:** 1 Department of Cardiology, Peking University China-Japan Friendship School of Clinical Medicine, Beijing, 100029 China; 2 Department of Cardiology, China-Japan Friendship Hospital, 2 Yinghua Dongjie, Chaoyang District, Beijing, 100029 China; 3 Graduate School of Peking Union Medical College, Chinese Academy of Medical Sciences and Peking Union Medical College, Beijing, 100029 China

**Keywords:** Cardiovascular disease, Diabetes, Chinese guideline, Predictive value, Adults

## Abstract

**Objective:**

Chinese diabetes society has published the new diagnostic criteria for diabetes in China (2020 edition). We aimed to investigate the predictive value of new diabetes-diagnosed criteria for cardiovascular diseases (CVD).

**Methods:**

A total of 5884 individuals from the China Health and Retirement Longitudinal Study in 2011 and 2018 were enrolled. Baseline characteristics and outcome data were compared. The association between diabetes diagnosed by two criteria and future CVD was identified by Kaplan–Meier curves, Cox regression analyses, and receiver-operating characteristic analyses. Delong’s test was conducted to compare the predictive value for future CVD between diabetes diagnosed by the 2020 edition and diabetes diagnosed by the previous version.

**Results:**

After multivariate adjustment, both diabetes diagnosed by the 2020 edition and diabetes diagnosed by the previous edition is associated with CVD (HR 1.607, 95% CI 1.221–2.115, *P* < 0.001; HR 1.244, 95% CI 1.060–1.460, *P* = 0.007, respectively). The Kaplan–Meier analysis indicated that diabetes patients have more cardiovascular risk (log-rank *P*<0.001). Moreover, diabetes diagnosed in the 2020 edition illustrated an area under the curve (AUC) of 0.673 for predicting CVD, while diabetes diagnosed in the previous edition showed an AUC of 0.638 (DeLong’s test *P*<0.01).

**Conclusion:**

Diabetes diagnosis criteria (2020 edition) in China had better performance in predicting cardiovascular diseases than the previous edition.

## Introduction

The prevalence of diabetes is still rising for decades, adding to the enormous burden of national finance. In 2013, a national and representative cross-sectional investigation of the detection of chronic diseases and their risk factors in China showed that the prevalence of diabetes in the Chinese population over 18 years old reached 10.3% [1]. In 2017–2018, the survey conducted by the Chinese diabetes society showed that the prevalence rate of diabetes in Chinese adults was as high as 11.2% [2], and there is still a large proportion of diabetes patients who have not been diagnosed and are not under standardized medical treatment [3].

As the standardization of glycated hemoglobin A1c testing is gradually improving in China [1, 4], the Chinese diabetes society has released the 2020 edition of diabetes prevention and treatment guidelines in China, indicating that HBA1C ≥ 6.5% can be used as the supplementary diagnostic standard for diabetes in medical institutions that adopt standardized testing methods and have strict quality control [5]. Diabetes is an independent predictor of cardiovascular disease, and cardiovascular disease is more common in people with diabetes by 2–3 times compared with the average population [6]. Whether the predictive value of diabetes diagnosed according to the new diagnostic criteria on cardiovascular events is higher than that of diabetes diagnosed according to the original diagnostic criteria is a crucial issue for promoting the new diagnostic criteria in China and has not been reported yet.

This study aimed to evaluate the predictive value of the new diabetic diagnostic criteria (2020 edition) for cardiovascular disease complications compared with the previous diagnostic criteria.

## Methods

### Study design and population

This cohort study was based on the data extracted from the China Health and Retirement Longitudinal Study (CHARLS), a large-scale longitudinal prospective cohort study in China [7]. The protocol of CHARLS has been described in detail elsewhere (http://charls.pku.edu.cn/index/en.html).

**Fig. 1:**
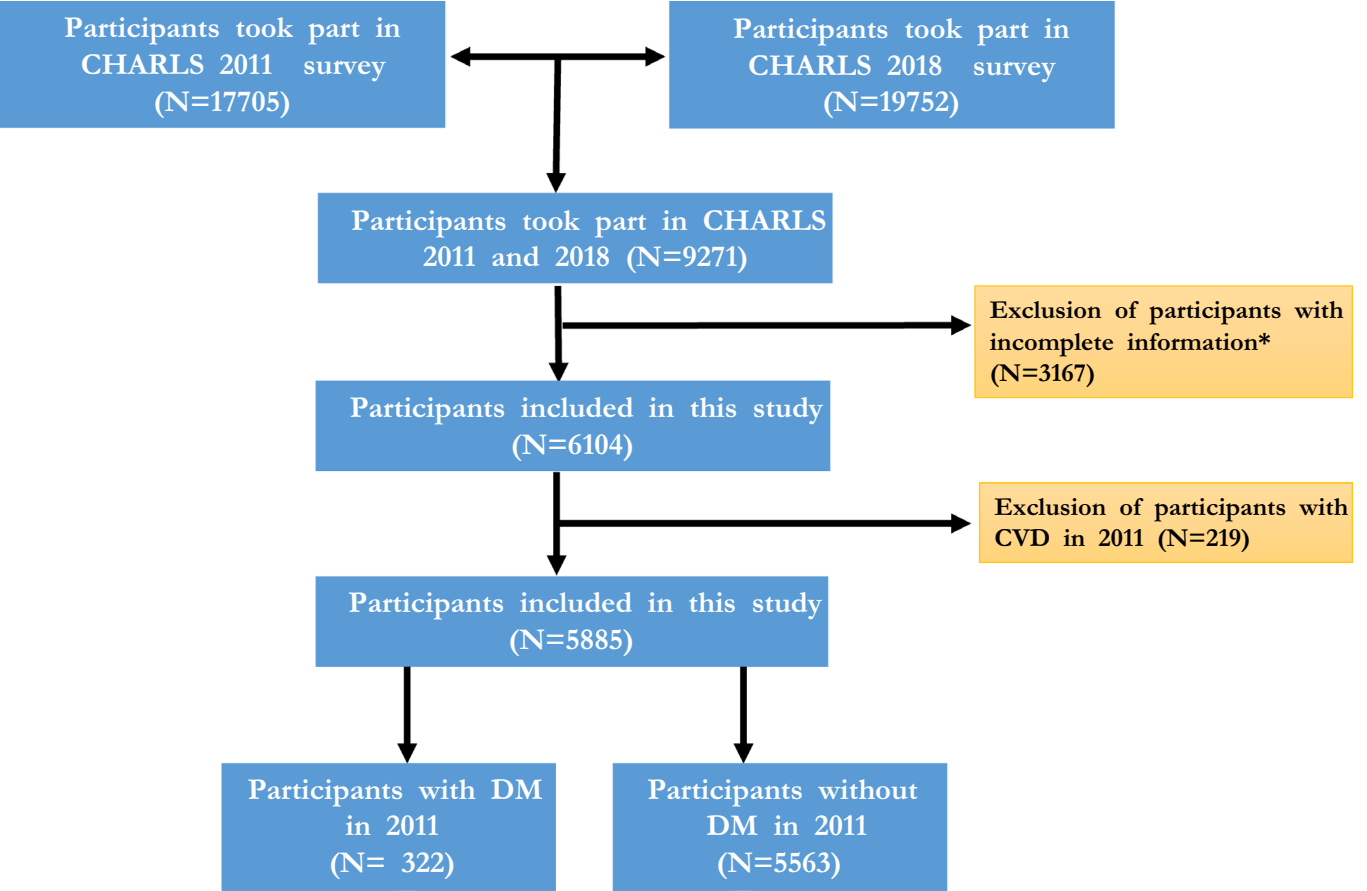
Study flowchart of the procedure extracting the participants from CHARLS. *Incomplete information includes age, sex, FBG, HbA1c, CVD condition, drugs information

In brief, 17,708 participants aged 45 years or above from 28 provinces in China were recruited by a four-stage stratified cluster sampling, adopting a multistage probability sampling technology in the first wave (W1) between 2011 and 2012 with a more than 80% response rate. The 9271 individuals were recruited with available follow-up data from the fourth wave (W4, 2018). All participants signed informed consent prior to participating. Participants with incomplete data on glycated hemoglobin in the blood test and information about diabetes, heart problem, and stroke were excluded, and participants with more than 10% missing data (Fig. 1). The institutional review board of Peking University approved the ethical review and experimental protocols of CHARLS (IRB00001052–11015).

### Data collection and definition

In the W1, a structured questionnaire was performed by researchers to acquire demographic status, including age, sex, educational level, smoking and drinking status, and health-related information. Essential health information included medical conditions (heart disease, hypertension, diabetes, stroke, and dyslipidemia) and medication treatment for heart problems, stroke, diabetes, hypertension, and dyslipidemia. Measurement of primary vital data such as height, weight, and diastolic and systolic blood pressure was acquired by trained nurses. The staff of the Chinese Center for Disease Control and Prevention was responsible for determining the biomarkers in venous blood samples placing at − 80 ℃ in a deep freezer in the CDC, such as glycated hemoglobin (HbA1c), total cholesterol (TC), high-density lipoprotein cholesterol (HDL-C), low-density lipoprotein cholesterol (LDL-C), triglycerides (TG), C-reactive protein (CRP), fast blood glucose, blood urea nitrogen (BUN), uric acid (UA), and creatinine. Boronate affinity and high-performance liquid chromatography (HPLC) was used for the HbA1c assay throughout the follow-up (W1–W4) under the daily quality control at the Youanmen Center for Clinical Laboratory of Capital Medical University.

Diabetes was diagnosed based on previous criteria (2017 guideline from the China Diabetes Society) as FBG>125 mg/dl in the blood test, self-reported diabetes diagnosed by a doctor, or taking treatment of antidiabetic medications. According to the 2020 edition guideline of diabetes mellitus in China from the China Diabetes Society, the new diagnostic criteria for diabetes were added to the previous standards with HbA1c > 6.5% in the blood test. The cardiovascular disease outcome was defined as a self-reported doctor-diagnosed heart disease (including coronary atherosclerosis, heart attack, heart failure, or other heart problems) or stroke during the follow-up period. Hypertension and dyslipidemia were defined as self-reported physician diagnoses of medical condition or being on medication.

### Statistical analysis

Continuous variables were expressed as mean (standard deviation, SD) or median (interquartile ranges, IQR) depending on normal distribution or not, while the number with percentage was presented for categorical variables. The diagnosis of diabetes classified all participants according to previous standard and 2020 guidelines, respectively. Student’s t-test or Mann–Whitney U test, and Chi-square test or Fisher’s exact test were conducted for continuous variables or categorical variables as appropriate. The univariate and multivariable Cox proportional regression analyses were conducted to estimate the relationship between diabetes and CVD. In Model 1, the Cox analysis was adjusted for age and gender. In Model 2, further adjustments of BMI, smoking, drinking, medical condition (including hypertension and dyslipidemia), laboratory markers (including WBC, TC, TG, HDL-C, LDL-C, CRP), and medication against hypertension and dyslipidemia were performed. The cumulative hazard ratio of cardiovascular diseases among diabetes and non-diabetes groups was exhibited by Kaplan–Meier analysis and compared using the log-rank test. To evaluate the difference in the incremental predictive value between diabetes diagnosed by the Chinese guideline 2020 edition and diabetes diagnosed by the previous standard, receiver operating characteristic curves (ROC) were conducted by combining diabetes 2020 edition and diabetes diagnosed by the last edition to traditional CVD risk factors based on multivariate Cox model 2 respectively and were compared by Delong’s test. R (version 4.1.3, Vienna, Austria) was performed for all statistical analyses. *P* < 0.05 was regarded as statistical significance (2-tailed test).

## Results

### Baseline characteristics

The 9271 individuals who took part in both W1 and W4 data investigation were included in this study. 3167 participants were excluded because of the missing data on health conditions and blood test sampling or died during the follow-up. Finally, 5884 individuals without CVD at baseline (W1) were recruited in this study (Fig. 1). The baseline characteristics are illustrated in Tables 1 and 2. The mean age of 5884 participants was 57.80 years. The previous standard diagnosed 230 diabetes participants, while the 2020 China guidelines diagnosed 322 diabetes participants. There were significant differences in BMI, WBC, TC, TG, HDL-C, LDL-C, and CRP among participants with or without diabetes diagnosed by 2020 guidelines (*P* > 0.05).

**Table 1:** The baseline characteristics of participants with or without diabetes diagnosed by 2020 edition

	Overall	Diabetes 2020 edition	Non-diabetes	*P*
n	5884	322	5562	
Age (years)	57.80 (9.01)	58.91 (8.51)	57.74 (9.04)	0.023
Male (%)	2634 (44.8)	131 (40.7)	2503 (45.0)	0.145
BMI	23.44 (3.77)	25.36 (4.42)	23.33 (3.70)	< 0.001
WBC (10^9/L)	6.21 (1.82)	6.65 (2.02)	6.19 (1.80)	< 0.001
Hemoglobin (g/dl)	14.33 (2.20)	14.44 (2.16)	14.32 (2.20)	0.373
BUN (mg/dL)	15.60 (4.35)	15.87 (4.62)	15.59 (4.34)	0.264
FBG (mg/dL)	108.86 (33.41)	177.33 (86.99)	104.89 (21.37)	< 0.001
Creatinine (mg/dL)	0.76 (0.18)	0.77 (0.22)	0.76 (0.17)	0.803
TC (mg/dL)	190.21 (167.40, 214.95)	198.52 (174.84, 223.36)	189.82 (167.01, 214.56)	< 0.001
TG (mg/dL)	103.54 (73.46, 150.45)	136.73 (92.04, 209.52)	101.78 (72.57, 147.79)	< 0.001
HDL-C (mg/dL)	49.87 (40.98, 60.31)	42.53 (35.57, 50.93)	50.26 (41.37, 60.70)	< 0.001
LDL-C (mg/dL)	114.05 (93.17, 136.86)	119.85 (97.04, 146.52)	113.85 (93.17, 136.47)	0.004
CRP (mg/dL)	0.95 (0.52, 1.97)	1.56 (0.82, 3.16)	0.92 (0.51, 1.91)	< 0.001
HbA1c (%)	5.10 (4.90, 5.40)	7.00 (5.50, 8.20)	5.00 (4.50, 5.30)	< 0.001
Hypertension (%)	1105 (18.8)	118 (36.6)	987 (17.7)	< 0.001
Dyslipidemia (%)	370 (6.3)	80 (24.8)	290 (5.2)	< 0.001
Kidney disease (%)	172 (2.9)	9 (2.8)	163 (2.9)	0.933
Smoking (%)	2196 (37.3)	112 (34.8)	2084 (37.5)	0.363
Drinking (%)	1997 (33.9)	97 (30.1)	1900 (34.2)	0.154
Anti-dyslipidemia (%)	283 (4.8)	61 (18.9)	222 (4.0)	0.012
Heart problem in W4 (%)	408 (6.9)	37 (11.5)	371 (6.7)	0.001
Stroke in W4 (%)	243 (4.1)	27 (8.4)	216 (3.9)	< 0.001
CVD in W4 (%)	612 (10.4)	60 (18.6)	552 (9.9)	< 0.001

**Table 2:** The baseline characteristics of participants with or without diabetes diagnosed by 2017 edition

	Overall	Diabetes	Non-diabetes	*P*
n	5884	230	5654	
Age (years)	57.80 (9.01)	59.06 (8.40)	57.75 (9.03)	0.031
Male (%)	2634 (44.8)	93 (40.4)	2541 (44.9)	0.201
BMI	23.44 (3.77)	25.28 (4.76)	23.37 (3.71)	< 0.001
WBC (10^9/L)	6.21 (1.82)	6.62 (2.01)	6.20 (1.81)	< 0.001
Hemoglobin (g/dL)	14.33 (2.20)	14.19 (2.12)	14.34 (2.21)	0.327
BUN (mg/dL)	15.60 (4.35)	15.88 (4.75)	15.59 (4.34)	0.32
FBG (mg/dL)	108.86 (33.41)	163.35 (77.66)	106.64 (28.13)	< 0.001
Creatinine (mg/dL)	0.76 (0.18)	0.77 (0.22)	0.76 (0.17)	0.526
TC (mg/dL)	190.21 (167.40, 214.95)	195.43 (172.04, 221.43)	189.82 (167.01, 214.56)	0.051
TG (mg/dL)	103.54 (73.46, 150.45)	130.54 (84.08, 202.22)	103.54 (72.57, 148.68)	< 0.001
HDL-C (mg/dL)	49.87 (40.98, 60.31)	43.49 (36.73, 51.03)	49.87 (40.98, 60.70)	< 0.001
LDL-C (mg/dL)	114.05 (93.17, 136.86)	118.49 (98.29, 144.01)	114.05 (93.17, 136.86)	0.024
CRP (mg/dL)	0.95 (0.52, 1.97)	1.54 (0.80, 3.13)	0.93 (0.51, 1.93)	< 0.001
HbA1c (%)	5.10 (4.90, 5.40)	6.05 (5.30, 7.70)	5.08 (4.80, 5.40)	< 0.001
Hypertension (%)	1105 (18.8)	95 (41.3)	1010 (17.9)	< 0.001
Dyslipidemia (%)	370 (6.3)	69 (30.0)	301 (5.3)	< 0.001
Kidney disease (%)	172 (2.9)	6 (2.6)	166 (2.9)	0.929
Smoking (%)	2196 (37.3)	75 (32.6)	2121 (37.5)	0.15
Drinking (%)	1997 (33.9)	63 (27.4)	1934 (34.2)	0.039
Anti-dyslipidemia (%)	283 (4.8)	52 (22.6)	231 (4.0)	0.008
Heart problem in W4 (%)	408 (6.9)	27 (11.7)	381 (6.7)	0.005
Stroke in W4 (%)	243 (4.1)	18 (7.8)	225 (4.0)	0.007
CVD in W4 (%)	612 (10.4)	42 (18.3)	570 (10.1)	< 0.001

### Future CVD risk

Over the 6.78 years of follow-up time, 60 of 322 diabetes (2020 edition) patients have developed the CVD, while 552 of 5562 non-diabetes (2020 edition) patients experienced CVD. Diabetes (2020 edition) patients had an increased incidence of CVD compared with non-diabetes patients (18.6% vs. 9.9% *P* < 0.001). At the same time, 42 of 230 diabetes (previous standard) patients experienced CVD during the follow-up period, while 570 of 5654 non-diabetes patients developed CVD. Similarly, the CVD incidence in diabetes groups was higher than in non-diabetes groups (18.2% vs. 10.0% *P* < 0.001).

**Fig. 2:**
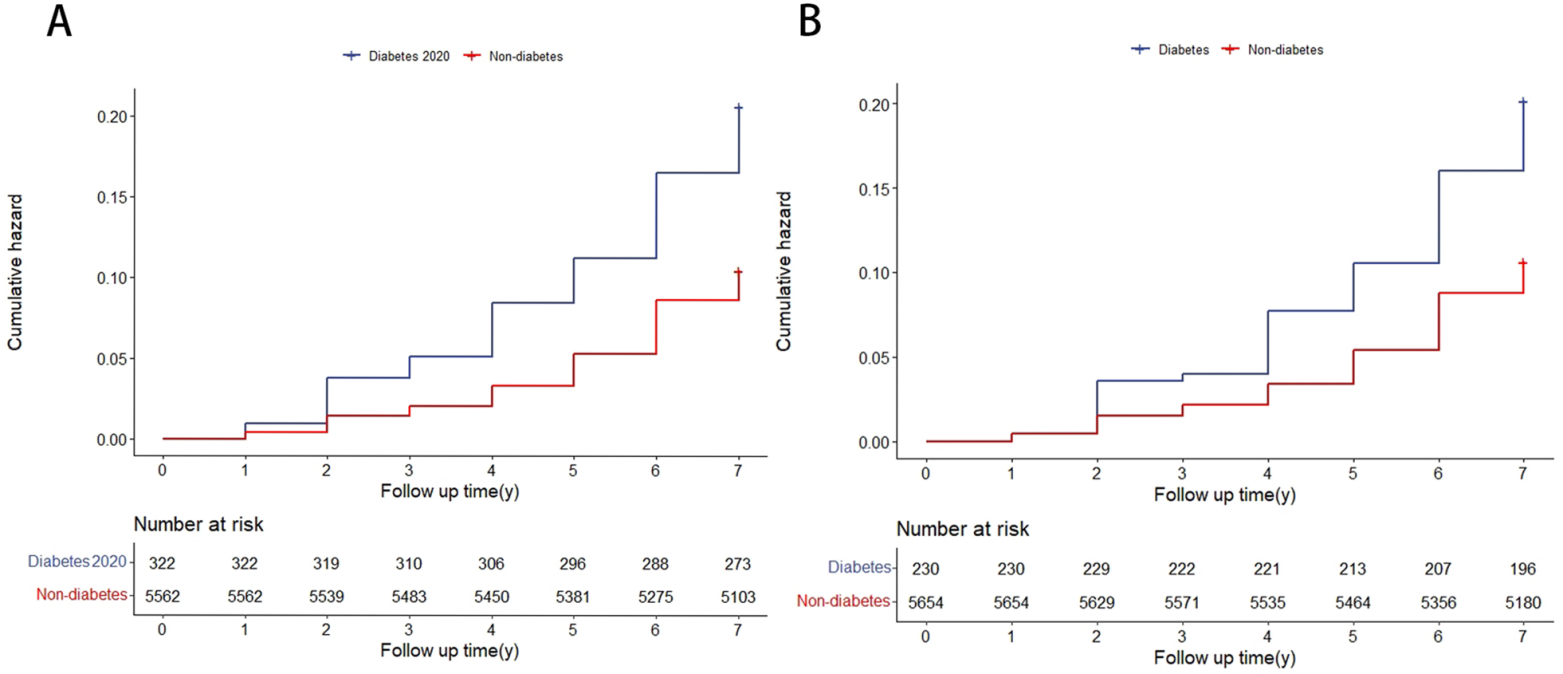
The results of Kaplan–Meier analysis of CVD in patients with and without diabetes showing the cumulative incidence of CVD in patients with and without diabetes diagnosed by 2020 edition (**A**) and 2017 edition (**B**), respectively

The Kaplan–Meier curves analyses exhibited that diabetes (2020 edition) groups had higher rates of CVD than non-diabetes groups with significant discrepancy (log-rank *P* < 0.001). Similarly, diabetes groups (previous standard) had higher CVD risk than non-diabetes groups shown by the cumulative hazard ratio (Fig. 2).

### Relationship between diabetes and CVD

According to Cox regression analysis, after adjusting for age and gender and complete multivariable adjustment, diabetes (2020 edition) was positively associated with the increased CVD risk (HR 1.894, 95% CI 1.451–2.473, *P* < 0.001; HR 1.607, 95% CI 1.221–2.115, *P* < 0.001, respectively). Diabetes diagnosed by the previous standard was positively related to future CVD risk based on multivariable Cox analyses (HR 1.244, 95%CI 1.060–1.460, *P* = 0.007) (Table 3).

**Table 3:** The Cox regression analyses showing the association between diabetes and cardiovascular disease

	Unadjusted	*P*	Model 1	*P*	Model 2	*P*
Diabetes (2017 edition)	1.884(1.377–2.577)	< 0.001	1.344(1.149–1.573)	< 0.001	1.244(1.060–1.460)	0.007
Diabetes (2020 edition)	1.967(1.507–2.568)	< 0.001	1.894(1.451–2.473)	< 0.001	1.607(1.221–2.115)	0.001

Model 1 adjusted for age and gender

Model 2 adjusted for age, gender, BMI, hypertension, dyslipidemia, smoking, drinking, WBC, TC, TG, HDL-C, LDL-C, CRP, Medication against hypertension and dyslipidemia

### Discrimination of predictive ability for CVD

**Fig. 3:**
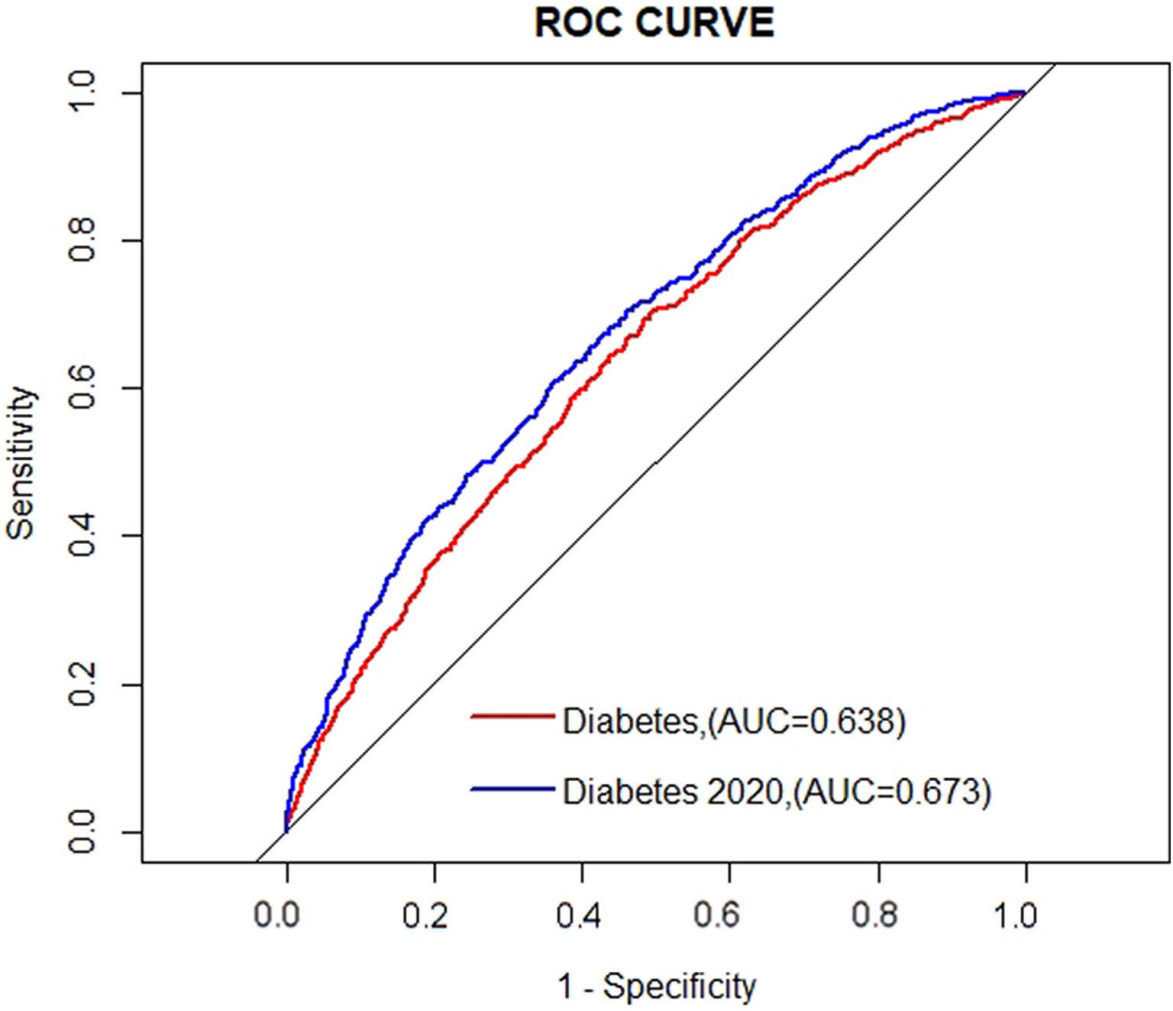
The comparison about the predictive value in CVD of diabetes diagnoses criteria 2017 edition and its 2020 edition. *AUC* area under the curve

The ROC result showed the discrimination of predictive ability between diabetes diagnosed by the 2020 edition and the previous one (Fig. 3). To be specific, the combined model which added diabetes (2020 edition) to traditional risk factors had a more accurate prediction of CVD (AUC 0.638) than the combined model adding diabetes (previous standard) (AUC 0.673), yielding an improvement in predictive value for future CVD (*P* < 0.01 by DeLong’s test).

## Discussion

This study compared the predictive ability for cardiovascular disease between the 2020 edition of diabetes prevention and treatment guidelines in China and previous diagnostic criteria based on CHARLS data. It was demonstrated that diabetes diagnosed by either the 2020 edition or the previous standard was positively associated with cardiovascular disease risk. The 2020 edition had better predictive value for the development of cardiovascular diseases highlighting the 2020 edition’s clinical utility.

The 2020 edition of diabetes prevention and treatment guidelines in China from the China Diabetes Society include glycated hemoglobin in the diagnostic criteria for diabetes, indicating that HbA1c ≥ 6.5% can be used as the supplementary diagnostic standard for diabetes in medical institutions that adopt standardized testing methods and have strict guidelines quality control [5]. In 2011, the World Health Organization (WHO) recommended using HbA1c to diagnose diabetes in countries where conditions are available, with a cut-off point of 6.5% [8]. However, due to the variability of HbA1c, accurate assays require an accredited laboratory with an external quality assurance strategy to conduct analysis [9]. In recent decades, with the standardization and unification of HbA1c testing standards in China [10], the Chinese diabetes society suggested that HbA1c can be the supplementary diabetes diagnostic standard with a cut-off of 6.5%, the same as WHO.

HbA1c levels reflect glucose exposure over the 120-day mean life of red blood cells [11]. The main advantage is that they are readily detectable without fasting, are not affected by diet or stress, and are more stable than glucose [12]. The primary function of HbA1c is an indicator of other glycosylated molecules, such as advanced glycosylated end products, which may be drivers of vascular inflammation and damage to blood vessels [13, 14].

HbA1c is regarded as the gold standard for predicting the risk of glucose-related vascular complications of diabetes within 5–10 years [15]. A prospective observational study reported that Hba1c significantly predicted all-cause mortality and cardiovascular disease, even below the diagnostic threshold for diabetes, independent of age and other risk factors in Europe [16]. A meta-analysis incorporating 46 studies established the optimal HbA1c levels in patients with diabetes are in the range of 6.0% to 8.0%, and those in people without diabetes are in the range of 5.0% to 6.0% [17]. Another meta-analysis reported that in patients with type 2 diabetes, each 1% increase in HbA1c levels was related to a 17% increase in cardiovascular events [18]. However, a recent retrospective study that enrolled about twenty thousand participants showed that higher HbA1c variability instead of high HbA1c level conferred a positive relationship to all-cause mortality and cardiovascular diseases of diabetes [19]. An RCT research illustrated that long-term HbA1c variability and HbA1c mean are associated with the increased risk of all-cause mortality [20]. Our results are broadly consistent with previous studies showing that the new diagnostic criteria of glycosylated hemoglobin included has a more predictive value of cardiovascular disease.

A high level of HbA1c indicates a state of poor glycemic control and insulin resistance [21]. Over time, long-term hyperglycemia can exacerbate inflammation, cause oxidative stress, aggravate endothelial dysfunction, enhance foam cell formation, and promote smooth muscle proliferation [22–25]. Atherosclerosis and vascular remodeling can also be caused by these pathophysiological changes, leading to cardiovascular disease [26]. Adding HbA1c to the diagnostic criteria for diabetes in China could improve the clinical significance of routinely measuring HbA1c levels in the average population and prevent the development of diabetes. Further, it can help estimate the risk of subclinical atherosclerosis [27] and subsequent CVD and monitor the impact of therapeutic interventions on this risk.

## Limitation

Some limitations in the present study should be acknowledged. First, the population included in CHARLS was adults older than 45. As a result, our conclusion may not apply to teenagers. However, an editorial reported that HbA1c should not be used to diagnose all children and teenagers [9]. Although diabetes among teenagers has increased, most diabetes patients in China are still adults. Second, no dynamic changes in HbA1c levels were recorded in the CHARLS study, which may also have clinical significance. Further, our study cannot explore the mechanism of HbA1c promoting the occurrence and development of cardiovascular disease, and the relevant agent needs to be further explored.

## Conclusion

Our study supported that diabetes was a risk factor for CVD incidence. The diabetes diagnoses criteria 2020 edition in China had a better predictive value for developing cardiovascular diseases than the previous edition.

## Data Availability

The data that support the findings of this study are available from the China Health and Retirement Longitudinal Study (CHARLS), but restrictions apply to the availability of these data, which were used under license for the current study, and so are not publicly available. Data are, however available from the author Zixiang Ye (yezxiang@yeah.net) upon reasonable request and with permission of CHARLS.

## Abbreviations

95%CI: 95% Confidence intervals
CVD: Cardiovascular disease
IR: Insulin resistance
TyG: Triglyceride glucose
MACE: Major adverse cardiovascular events
CHARLS: China Health, and Retirement Longitudinal Study
TG: Triglycerides
FBG: Fast blood glucose
TC: Total cholesterol
hsCRP: High-sensitivity C-reactive protein
HDL-C: High-density lipoprotein cholesterol
LDL-C: Low-density lipoprotein cholesterol
BUN: Blood urea nitrogen
DM: Diabetes mellitus
eGFR: Estimated glomerular filtration
CKD: Chronic kidney disease
IQR: Interquartile ranges
SD: Standard deviation
CAD: Coronary artery diseases
PCI: Percutaneous coronary intervention
RCT: Randomized controlled trial
HPLC: High-performance liquid chromatography
